# Iron dyshomeostasis and time-course changes in iron-uptake systems and ferritin level in relation to pro-inflammatory microglia polarization in sepsis-induced encephalopathy

**DOI:** 10.3389/fphys.2022.953206

**Published:** 2022-08-12

**Authors:** Nivin Sharawy, Ahmad Abdel-Aliem Imam, Basma Emad Aboulhoda, Mohamed Mansour Khalifa, George N. B. Morcos, Waleed Ahmed Abd Algaleel, Passant E. Moustafa, Marwan A. Abdelbaset, Tarek Shoukry

**Affiliations:** ^1^ Department of Physiology, Faculty of Medicine, Cairo University, Cairo, Egypt; ^2^ Preclinical Sciences, College of Osteopathic Medicine, William Carey University, Hattiesburg, MS, United States; ^3^ Faculty of Medicine, Cairo University, Cairo, Egypt; ^4^ Department of Anatomy and Embryology, Faculty of Medicine, Cairo University, Cairo, Egypt; ^5^ Department of Human Physiology, College of Medicine, King Saud University, Riyadh, Saudi Arabia; ^6^ Department of Biochemistry, Faculty of Medicine, Cairo University, Cairo, Egypt; ^7^ Department of Basic Medical Sciences, Faculty of Medicine, King Salman International University, El-Tor, Egypt; ^8^ Department of Pharmacology, National Research Center, Cairo, Egypt

**Keywords:** brain, microglia, DMT1, ZIP, CUBILIN

## Abstract

Encephalopathy is a frequent and lethal consequence of sepsis. Recently, a growing body of evidence has provided important insights into the role of iron dyshomeostasis in the context of inflammation. The molecular mechanisms underlying iron dyshomeostasis and its relationship with macrophage phenotypes are largely unknown. Here, we aimed to characterize the changes in iron-transporter and storage proteins and the microglia phenotype that occur during the course of sepsis, as well as their relationship with sepsis-induced encephalopathy. We used a cecal ligation and puncture (CLP) murine model that closely resembles sepsis-induced encephalopathy. Rats were subjected to CLP or sham laparotomy, then were neurologically assessed at 6 h, 24 h, and 3 days after sepsis induction. The serum and brain were collected for subsequent biochemical, histological, and immunohistochemical assessment. Here, an iron excess was observed at time points that followed the pro-inflammatory macrophage polarization in CLP-induced encephalopathy. Our results revealed that the upregulation of non-transferrin-bound iron uptake (NTBI) and ferritin reduction appeared to be partially responsible for the excess free iron detected within the brain tissues. We further demonstrated that the microglia were shifted toward the pro-inflammatory phenotype, leading to persistent neuro-inflammation and neuronal damage after CLP. Taken together, these findings led us to conclude that sepsis increased the susceptibility of the brain to the iron burden *via* the upregulation of NTBI and the reduction of ferritin, which was concomitantly and correlatively associated with dominance of pro-inflammatory microglia and could explain the neurological dysfunction observed during sepsis.

## Introduction

Sepsis is characterized by a biphasic immune response involving a hyper- and a hypo-inflammatory state, resulting in organ dysfunction, including brain dysfunction (Scindia et al., 2019; Michels et al., 2020; Sn et al., 2021). The dynamic changes in macrophage activation that occur during sepsis and the alterations in iron homeostasis related to differential macrophage responses during this condition have not been fully explored.

In the brain, iron is stored in glial cells and neurons. Iron overload generates reactive oxygen species *via* its participation in the Fenton reaction. This eventually leads to lipid, protein, and DNA damage (Lan et al., 2018). Tissue iron homeostasis can be tightly-regulated by the iron regulatory proteins that control iron uptake and storage. Two uptake systems that facilitate iron transport have recently been proposed, namely, the transferrin (TBI; composed of the transferrin receptor protein 1 (TfR-1) and cubilin) system and the NTBI; composed of the divalent metal transporter 1 (DMT-1) and zinc transporters (ZIPs)) system (Weiss et al., 2018). Iron is stored intracellularly as ferritin, which in turn mitigates the toxicity of free iron (Liu et al., 2019); (Liu et al., 2019). Moreover, the iron status seems to determine the type and intensity of inflammatory responses; however, these findings remain controversial. Although some reports showed that iron depletion promoted the pro-inflammatory response (Daglas and Adlard, 2018), other studies reported that iron overload was associated with exacerbation of the pro-inflammatory response (Fan et al., 2014).

Macrophages are believed to be the cardinal modulators of brain function. In addition, they remain dynamic, changing from the pro-inflammatory M1 to the anti-inflammatory M2 phenotypes, which show obvious differences in their gene expression signature and functions; i.e., the M1 phenotype contributes to brain degeneration by releasing pro-inflammatory mediators, whereas neuronal regeneration has been linked to protective factors produced by the M2 phenotype (Agoro et al., 2018; Hu et al., 2015). Macrophages have also been shown to be crucial for iron regulation (Daglas and Adlard, 2018). Moreover, *in vitro* studies revealed that the alteration of the cellular iron balance may be a consequence of macrophage polarization (Corna et al., 2010; Recalcati et al., 2019). Finally, controversial evidence demonstrated that iron overload could either dampen or exaggerate the inflammatory response (Pagani et al., 2011; Hoeft et al., 2017; Agoro et al., 2018; Zanganeh et al., 2016).

In this study, we aimed to investigate whether sepsis renders the brain more susceptible to iron overload by changing the transcription of iron transport genes and ferritin levels, and whether these events are associated with changes in the dynamics of macrophage activation.

## Materials and methods

### Animals

The animals were purchased from the National Research Centre, Cairo, Egypt. All experimental procedures were performed after approval was obtained from our institutional animal ethical committee (protocol number: CU-II-F11-20). Male albino rats weighing 160–200 g were randomly submitted to sham operation or CLP. Albino rats were housed at five animals per stainless steel cage (27 cm × 38 cm × 17 cm), fed *ad libitum*, and maintained in a room at a constant temperature and humidity with a 12 h light/12 h dark cycle.

### Cecal ligation and puncture model

The CLP surgery was performed as described previously (Gamal et al., 2018). Briefly, the rats were anesthetized with isoflurane and the cecum was exposed *via* a 3-cm midline incision, followed by ligation using a 3.0 silk suture at approximately 1 cm from the ileocecal valve. Subsequently, the cecum was perforated three times using a 16-gage needle. A small amount of feces was extruded and the cecum was returned to the peritoneal cavity. The skin incision was sutured with 4.0 silk suture. The animals were resuscitated using subcutaneous pre-warmed saline (1 ml) and received analgesia. Sham-operated rats were subjected to the same procedure with the exception that the cecum was neither ligated nor punctured. All procedures were performed by the same investigator.

The severity of sepsis was scored using a murine sepsis score (MSS), as described previously (Zhang et al., 2017; Shrum et al., 2014). At pre-set time points (6 h, 24 h, and 3 days postoperatively), the appearance, level of consciousness, spontaneous activity, response to stimuli, openness of the eyes, posture, and degree of labored breathing variables were included in the MSS (Supplementary File S1). Each variable was scored from 0 to 4 and a total score was calculated by two investigators who were blinded to the experiment.

The rats were sacrificed at 6 h, 24 h, and 3 days after CLP or sham surgery. The brain and serum were collected and stored at −80°C for biochemical analysis. Brain samples were also collected for histological analysis after trans-cranial perfusion with phosphate-buffered saline followed by 4% paraformaldehyde (PFA).

### Behavioral and sensorimotor analysis

All tests were performed prior to surgery and at 6 h, 24 h, and 3 days post-surgery. Moreover, all behavioral tests were performed at 10 am. For all tests, the rats were first acclimated for 30 min to the testing room. They were also trained before surgery to obtain an optimal level of performance.

### Accelerating rotarod test

A rotarod apparatus (Model No. 7750; Ugo Basile) was used as described previously (Gamal et al., 2015). Rats were placed on a stationary rod for 5 min; subsequently, the speed of rotation was accelerated smoothly from 0 to 20 rotations per min (rpm) over 5 min. Each rat was tested for the ability to remain on the rod. The latency to fall was measured three times using a stopwatch and the average was calculated for each rat; a shorter latency to fall was considered as a motor coordination dysfunction.

### Activity cage

A grid-floor activity cage (Model No. 7430, Ugo-Basile, Comerio, Italy) was used to measure locomotor activity. Each rat was placed individually in the activity chambers in a quiet room for 5 min. The beam-interruption information was processed and the total horizontal movements were recorded by the activity cage software. At the end of the 5-min period, the rat was removed and returned to its home cage.

### Novel object recognition

A NOR test was used to evaluate memory and exploratory behavior as reported previously (Tao et al., 2020; SN et al., 2021). The NOR test was performed in an arena formed by a white base surrounded by black walls (60 cm long × 40 cm wide × 40 cm high). The NOR test consisted of three phases: the habituation, familiarization, and test phases. Each rat was individually habituated to the testing arena once a day for three consecutive days (10 min per day). The familiarization session began 24 h after the last habituation session. Two identical objects were placed at a distance of 10 cm from the walls, and each rat was allowed to examine the objects for 5 min. The rat was then removed and placed back in its home cage for 30 min. Subsequently, one of the objects was replaced with a novel object. Each rat was allowed to explore the familiar and novel objects for 5 min, and the time each rat spent engaging in this activity was recorded by stop watch. Exploratory behavior was considered to have occurred when a rat sniffed or touched the object with one or both forepaws. The exploration index was obtained by subtracting the exploration time of the familiar object from that of the novel object divided by the total exploration time.

### Elevated body swing

This test was used to assess asymmetric motor behavior. Each rat was held by its tail at a height of 10 cm above the testing surface. Swinging was defined as turning the upper body by >10° to either side. The number and direction of swings was recorded three times in 10 trials. The percentage of the swing to the right was calculated, and the average scores were determined for each rat (Bruschetta et al., 2017; Tabuse et al., 2010).

### The adhesive removal

This test integrates sensorimotor feedback. Initially, two pieces of round stickers (1-cm diameter) were placed with equal pressure on each animal paw. The rats were then immediately returned to the test box. Then, the time taken by each rat to react to the presence of the adhesive tape (contact time) and the time it took to remove the two adhesives (removal time) were recorded using two stopwatches (Tabuse et al., 2010; Bouet et al., 2009).

### Real-time RT-PCR analysis

The TRIzol® reagent (Invitrogen) was used to extract total RNA from brain tissues (Livak, & Schmittgen, 2001). Spectrophotometry was used to quantify the extracted RNA or this procedure, 1 µg of purified RNA was reverse transcribed to double-stranded cDNA, which was then subjected to 30 cycles of PCR amplification in the presence of a primer targeting the *NP*, *NS1*, or *GAPDH* genes (Table 1). For normalization, *GAPDH* was used as the housekeeping gene. The relative mRNA expression levels were obtained using the ΔΔCt method.

**TABLE 1:** Sequences of primers used in real-time polymerase chain reaction (Real-time PCR).

Gene	Primer sequences (5′-3′)	
iNOS	Forward	5′- acc​caa​ggt​cta​cgt​tca​gg-3′
	Reverse	5′- cgc​aca​tct​ccg​caa​atg​ta-3′
Arg-1	Forward	5′- aca​aga​cag​ggc​tcc​ttt​ca-3′
	Reverse	5′- agc​aag​cca​agg​tta​aag​cc-3′
FIZZ	Forward	5′- atg​aac​aga​tgg​gcc​tcc​tg-3′
	Reverse	5′- ccc​aag​atc​cac​agg​caa​ag-3′
Cubilin	Forward	5′- AAT​GGA​TGT​GTG​CAG​CTC​AG-3′
	Reverse	5′- GGG​GTT​GCT​CAA​ACA​CTC​AT-3′
DMT1	Forward	5′- CAGTGCTCT GTA​CGT​AAC​CTG​TAA​GC-3′
	Reverse	5-′ CGC AGA AGAACG AGG ACC AA-3′
TfR1	Forward	5′- CTA​GTA​TCT​TGA​GGT​GGG​AGG​AAG​AG-3′
	Reverse	5′- GAG AAT CCC AGT GAG GGT CAG A-3′
ZIP8	Forward	5′- TGG TTG CAC CCC TCA CAA AT-3′
	Reverse	5-′ CACATGGTGCAC TGAAACCG-3′
GAPDH	Forward	5′- CCT GGA GAA ACC TGCCAA GTA T-3′
	Reverse	5-′ AGC CCA GGA TGC CCT TTA GT-3′

### Enzyme-linked immunosorbent assay

The brain tissue was lysed and homogenized using RIPA buffer (Sigma-Aldrich). The protein content of the lysates and serum was assayed using a BCA kit according to the manufacturer’s protocol (Pfeiffer and Looker, 2017). Antibodies specific for transferrin and ferritin were pre-coated onto a microplate, and the standards and samples were pipetted into the wells. The plates were washed and the reaction was stopped by the addition of Sulfuric acid. The absorbance at 450 nm was measured using a microplate reader, and the intensity of the color was proportional to the amount of ferritin and transferrin bound to the capture antibody.

### Serum and tissue iron parameter analysis

The Iron and Iron-Binding Capacity (TIBC) ELISA Kits (Abcam, Toronto, Canada) were used to measure serum iron levels and transferrin saturation as previously described (Pfeiffer and Looker, 2017). For this, 50 μl of TIBC Assay Buffer was added to 10–50 μl of serum/well, and was incubated for 10 min at 37°C. TIBC detector and developer solutions were added and incubated for 10 min at 37°C. Serum iron and TIBC were assayed by absorbance at an OD of 570 nm for standards and samples. The percentage of transferrin saturation was determined by dividing the total iron by the TIBC × 100. To measure brain iron concentration, the brain from each rat was dried and digested by acid. The tissue iron content was colorimetrically-determined.

### Histological study

Brain specimens from each animal were fixed in 10% saline formalin and processed to prepare 5-μm-thick paraffin sections for hematoxylin and eosin (H&E) staining. The extent of microgliosis and neuronal degeneration was graded in different brain regions as described previously (CH et al., 2018), where 0 indicates no significant lesion, 1 denotes minimal lesion (<1%), 2 indicates mild lesions (1%–25%), 3 denotes moderate lesion (26%–50%), 4 indicates marked affection (51%–75%), and 5 signifies severe/high-level lesion (76%–100%).

### Immunohistochemical analysis

Immunohistochemical assessment of the macrophage phenotype markers CD8, CD68, and CD 163 and the microglial marker Iba-1 was performed using the avidin–biotin peroxidase method as described previously (Magdy et al., 2021). Briefly, paraffin-embedded brain sections were dewaxed in xylene, rehydrated in a decreasing alcohol gradient, and microwave-treated (0.01 M trisodium citrate), for antigen retrieval. Quenching of the endogenous peroxidase activity was achieved *via* incubation in 10% Hydrogen peroxide. The sections where then incubated with the primary antibodies, as follows: anti-CD8 antibody (Thermo Fisher Scientific, MA5-13473, 1:25), rabbit polyclonal anti-ionized calcium-binding adapter molecule 1 (IBA1) antibody (ab153696, 1:500, Abcam®), rabbit monoclonal anti-CD86 antibody [C86/2160R] (ab234401, 1:50, IHC-P, Abcam®), and anti-CD163 antibody [EPR19518] (ab182422) (1:100, Abcam®, Cambridge, MA, United States). Staining was completed by incubation with the substrate-chromogen 3,3′- diaminobenzidine (DAB) and counterstaining with hematoxylin. The mouse spleen tissue was used as the positive control, whereas the negative controls were obtained through omission of the primary antibodies in the automated staining protocol.

### Statistical analysis

Molecular and neurological data were analyzed using the Graph Pad Prism software. A two-way ANOVA followed by the Bonferroni post-hoc test was performed to assess the significance of the differences detected between the groups. Data are presented as the mean ± S.E.M. Pearson’s correlations were used to explore the relationship between macrophage phenotype markers and iron levels within the brain tissues. For all comparisons, significance was set at *p* < 0.05.

## Results

### Sepsis causes the impairment of cognitive, behavioral, and motor coordination functions

**FIGURE 1:**
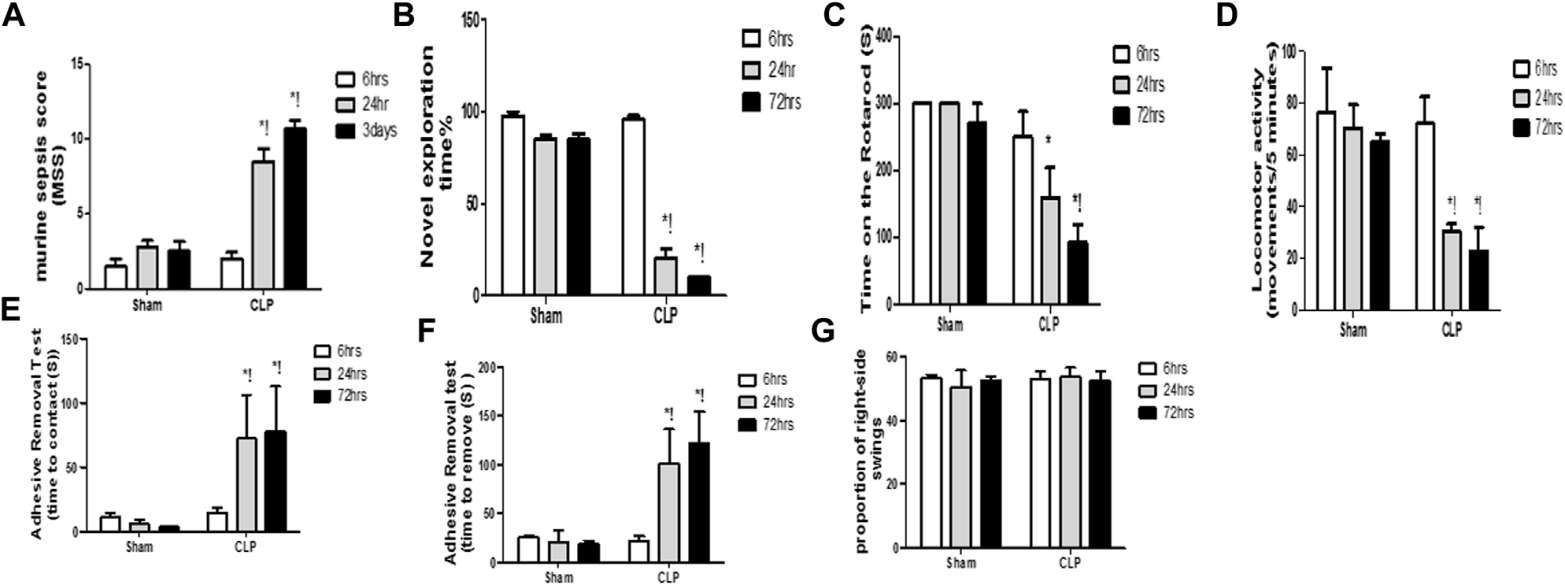
Altered cognitive, behavioral and motor coordination functions with sepsis. Murine sepsis score (MSS) **(A)** is significantly-induced by CLP. CLP decreased the percentage of the novel object exploration time **(B)**, the time spent on the rotarod **(C)**, and the locomotor activity **(D)** at 6, 24, and 72 h post-sepsis. The time for contact with **(E)** and removal of **(F)** the tape was significantly increased at 24 and 72 h after CLP. An asymmetrical motor behavior could not be observed after the elevated body swing test **(G)**. Data are the means ± SEM. *p < 0.05 vs. the corresponding sham group; !*p* < 0.05 vs. the 6 h CLP group; and ^#^ *p* < 0.05 vs. the 24 h CLP group. *N* = 10 rats per group.

Systemic insults, such as inflammation, can contribute to substantial and persistent brain insults, which result in functional disabilities. Sepsis was confirmed using the murine sepsis score (MSS), which evaluated the level of consciousness, spontaneous activity, response to stimuli, eye openness, posture, appearance, and the degree of labored breathing (Figure 1A).

The diagnosis of brain dysfunction essentially relied on the fundamental neurological, cognitive, and behavioural tests, including those that assessed the recognition function, motor co-ordination function, locomotor activity, asymmetric motor behaviour, and sensorimotor feedback. The current study evaluated cognitive function and motor coordination function *via* the novel object exploration test and accelerating rotarod test, respectively. We assessed the locomotor activity and asymmetric motor behaviors through the activity cage and elevated body swing test, respectively. The sensorimotor feedback was estimated through the adhesive removal test. Observational changes in the estimated parameters revealed significant decrease in the novel object exploration index, the time spent on the rotarod and the locomotor activity at 6 h, 24 h, and 3 days following CLP (Figures 1B–D).

This finding elucidated the association between septic-induced brain dysfunction and the neurologic sequelae, including the functional and cognitive decline. In the adhesive removal test, animals in the CLP group required significantly greater time to contact and remove the tape, whereas rats in all sham groups were able to contact and remove the tape simultaneously (Figures 1E,F). We did not observe laterality on the elevated body swing test in any of the rats treated with CLP or sham operation (Figure 1G), thereby confirming sepsis-induced behavioral and sensorimotor impairments.

### Sepsis induces encephalopathy by inducing neurodegeneration and cerebral micro-gliosis

The pathophysiology of sepsis-induced encephalopathy is a complex multifactorial process that can induce significant alterations in several vulnerable areas of the brain. The current study has, thus, evaluated the histological architecture of the cerebral cortex, the thalamus, the striatum, the cerebellum, the hippocampus and the dentate gyrus for the purpose of relating the functional changes to relevant structural alterations. Interestingly, this histopathological study has presented neurodegeneration, microglial activation and Blood brain barrier (BBB) impairment as plausible causes for the afore-mentioned cognitive, behavioral and locomotor dysfunctions. Those brain regions exhibited features of neuronal degeneration in the form of numerous dark neurons that have either shrunken, disfigured, irregular nuclei with extensively vacuolated cytoplasm or pyknotic, deeply-stained apoptotic nuclei.

Features of meningeal congestion were also observed in the cerebral cortex 24 h after induction of sepsis. The blood capillaries also exhibited interruption of their endothelial lining with widening of the perivascular Virchow Robin’s space. Those findings, collectively, represent a neuropathological basis for possible BBB dysfunction.

**FIGURE 2:**
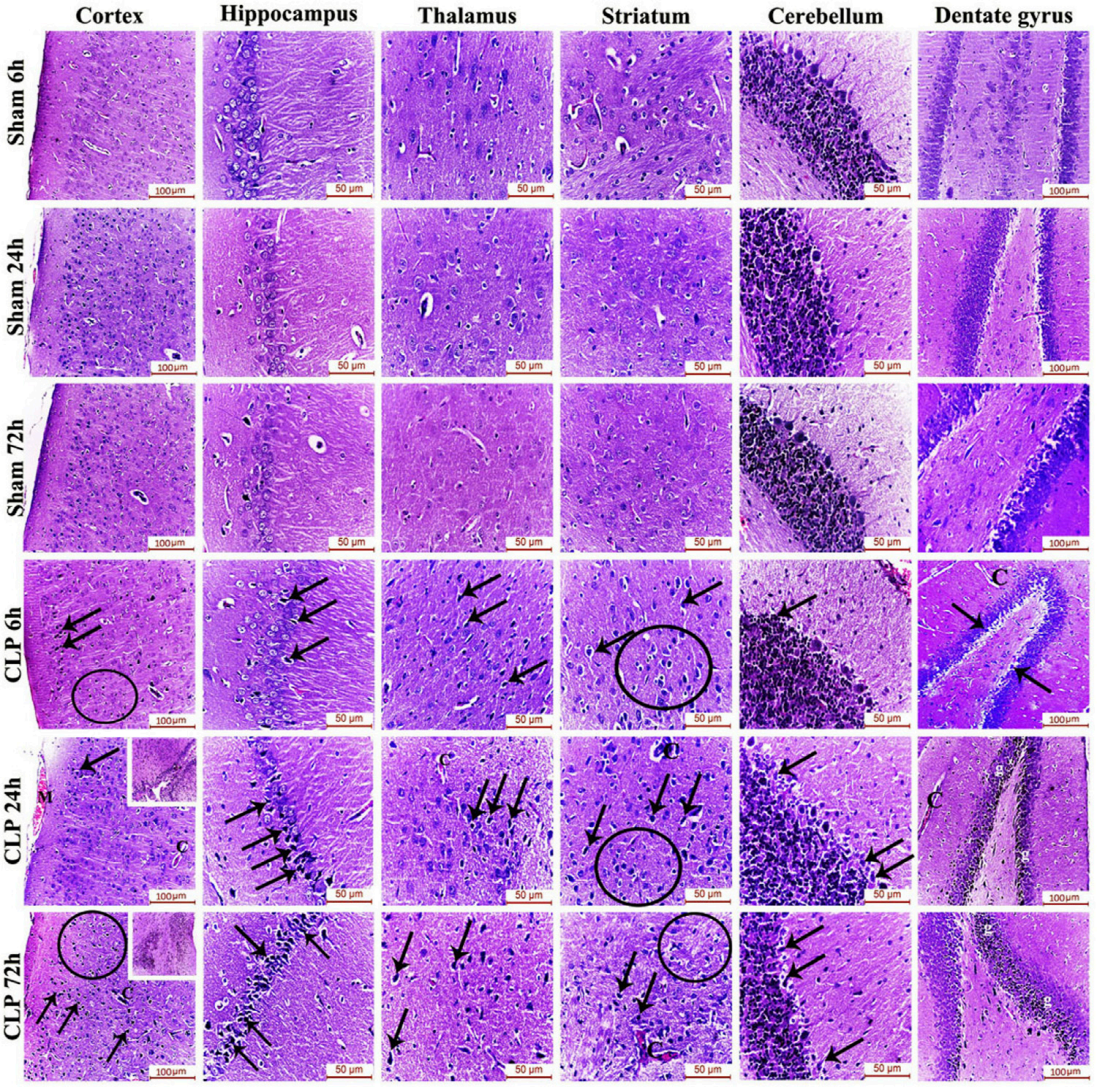
Sepsis-induced encephalopathy enhanced microgliosis and neuronal degeneration. H&E-stained sections of the different brain regions showing darkly-degenerated, apoptotic neurons (arrows) and glial cell proliferation (circles). The dentate gyrus shows marked microgliosis (g). The insets in the cerebral cortex show a picture of subependymal microgliosis in the CLP 24 h groups, and foci of focal cortical microgliosis in the CLP 72 h groups. Meningeal congestion (M) is also observed in the cerebral cortex of the CLP 24 h group. The blood capillaries (C) exhibit interruption of their endothelial lining with widening of Virchow Robin’s (perivascular) space.

The cerebral cortex and the dentate gyrus in particular have shown pronounced features of gliosis which were represented as subependymal microgliosis in the CLP 24 h groups, foci of focal cortical microglial proliferation in the CLP 72 h groups and marked microgliosis of the dentate gyrus in both the CLP 24 and 72 h groups (Figure 2; Table 2).

**TABLE 2:** H&E scoring of the cerebral cortex, hippocampus, thalamus, striatum, cerebellum, and dentate gyrus after 6, 24, and 72 h of subjection to sham and cecal ligation and puncture (CLP).

		Sham			CLP		
		6 h	24 h	72 h	6 h	24 h	72 h
Cerebral cortex	Microgliosis	0	0	0	2	4	4
	Neuronal degeneration	0	0	0	1	2	3
Hippocampus	Microgliosis	0	0	0	1	2	2
	Neuronal degeneration	0	0	0	1	2	3
Thalamus	Microgliosis	0	0	0	1	2	3
	Neuronal degeneration	0	0	0	1	1	1
Striatum	Microgliosis	0	0	0	1	2	3
	Neuronal degeneration	0	0	0	2	2	2
Cerebellum	Microgliosis	0	0	0	1	2	3
	Neuronal degeneration	0	0	0	1	1	1
Dentate gyrus	Microgliosis	0	0	0	1	3	3
	Neuronal degeneration	0	0	0	1	1	1

### Microglia exhibit a distinct activation phenotype in sepsis-induced encephalopathy

**FIGURE 3:**
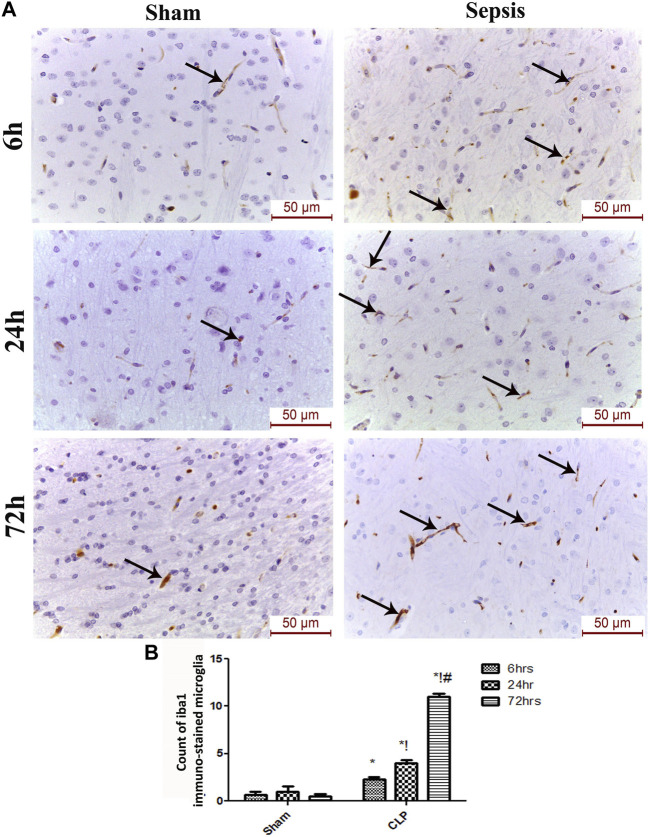
Sepsis aggravated the activation of microglia. **(A)** Immunohistochemical evaluation of Iba1 expression in the sham and cecal puncture and ligation (CLP) groups at different time points. **(B)** Count of Iba1 immunohistochemically stained microglia. Data are presented as the mean ± S.E.M. (*n* = 6). *: Statistically significant vs. the Sham; !:significant vs. 6 h CLP; #: significant vs. 24 h CLP using ANOVA with the Bonferroni post-hoc test.

The features of microgliosis observed in the histological study were further confirmed by the microglia activation marker, Iba-1, which was significantly increased at 6, 24, and 72 h after CLP (Figures 3A,B).

**FIGURE 4:**
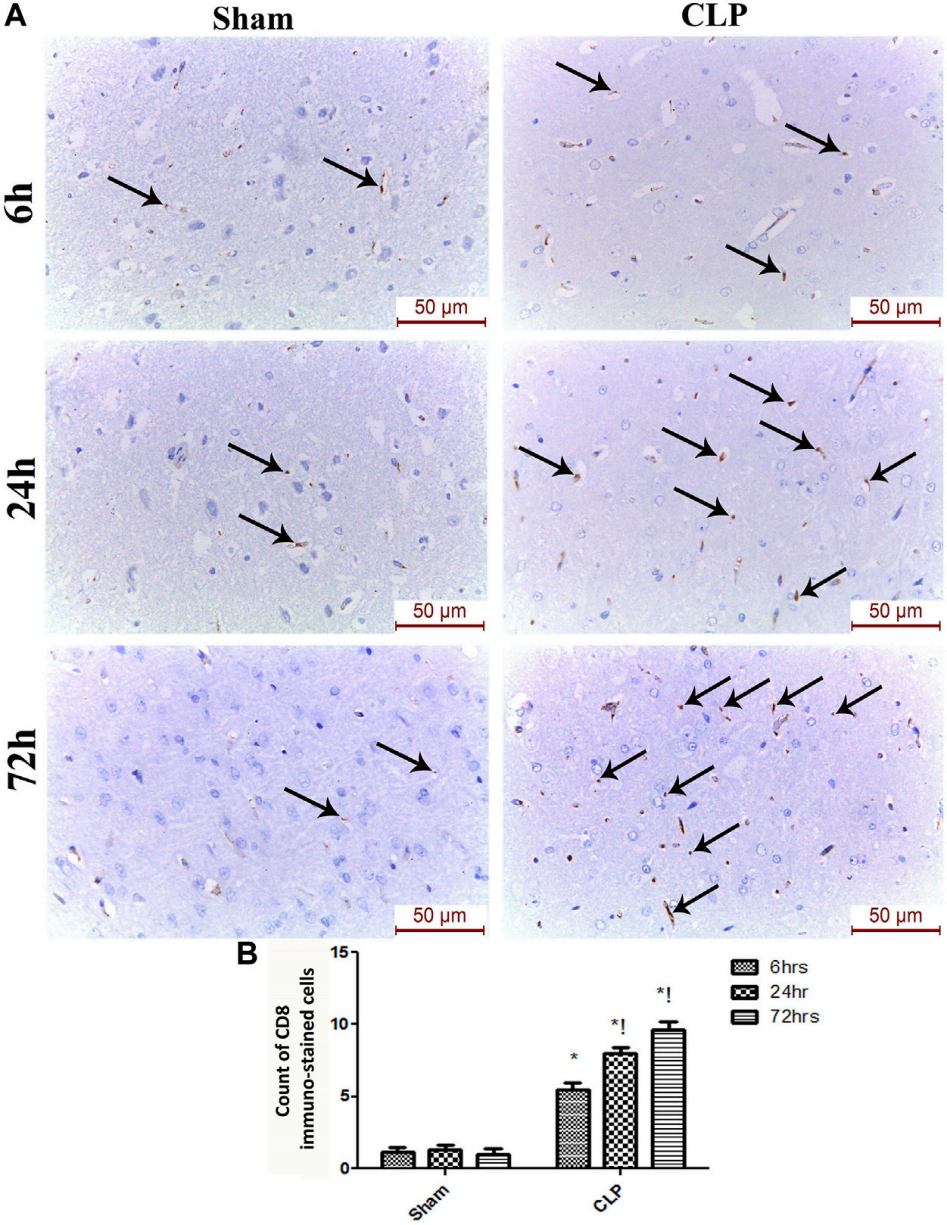
Sepsis enhanced the infiltration of CD 8 macrophages in brain tissues. **(A)** Immunohistochemical evaluation of CD8 expression in the sham and cecal puncture and ligation (CLP) groups at different time points. **(B)** Count of cells that were immunohistochemically stained for CD8. Data are presented as the mean ± S.E.M. (*n* = 6).*: Statistically significant vs. Sham; !: significant vs. 6 h CLP; #: significant vs. 24 h CLP; as assessed using ANOVA with the Bonferroni post-hoc test.

**FIGURE 5:**
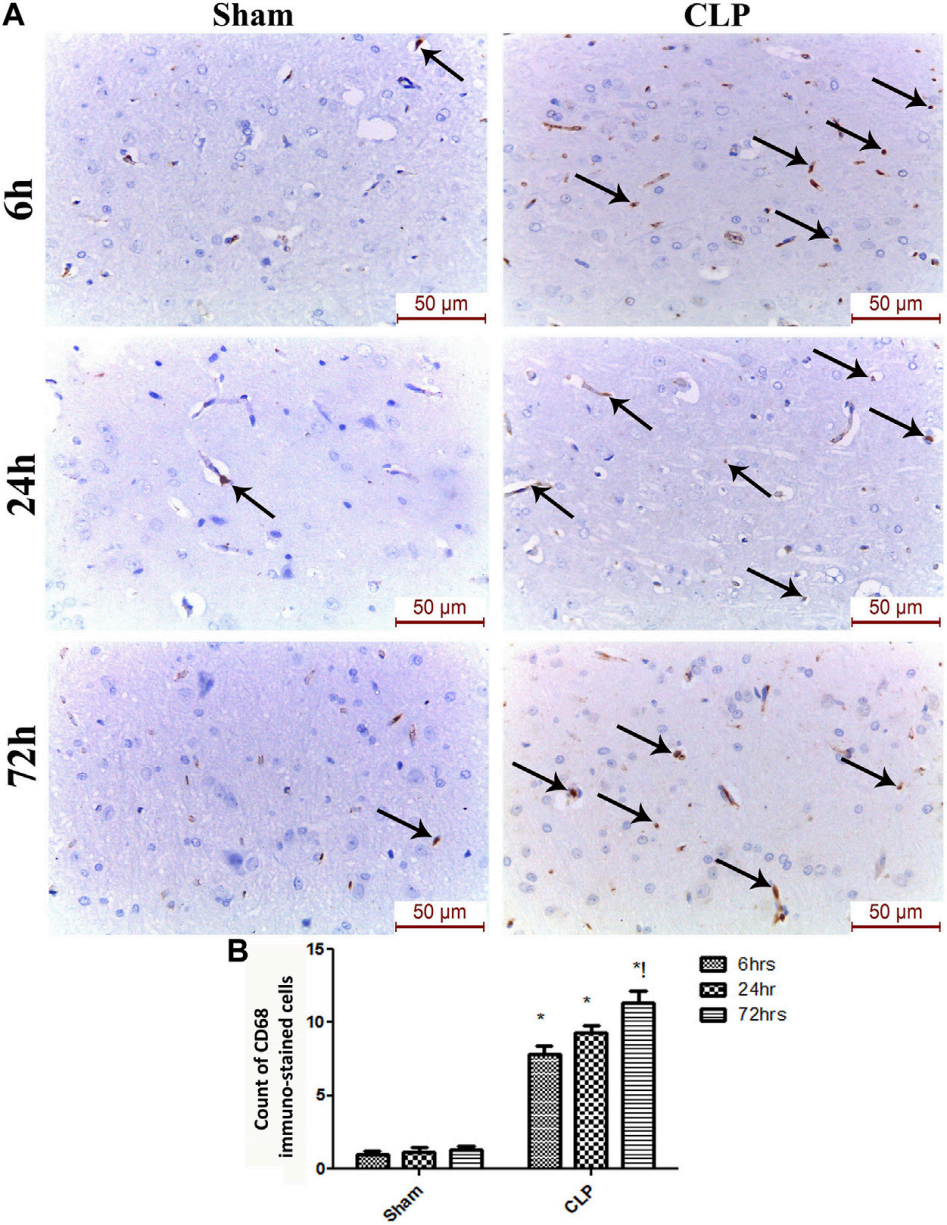
Sepsis enhanced the infiltration of CD 68 macrophages in brain tissues. **(A)** Representative images of CD68 immunohistochemical staining in the sham and cecal puncture and ligation (CLP) groups at different time points. **(B)** Count of cells that were immunohistochemically stained for CD68. Data are presented as the mean ± S.E.M. (*n* = 6). *: Statistically significant vs. the Sham; !: significant vs. 6 h CLP; #: significant vs. 24 h CLP; as assessed using ANOVA with the Bonferroni post-hoc test.

**FIGURE 6:**
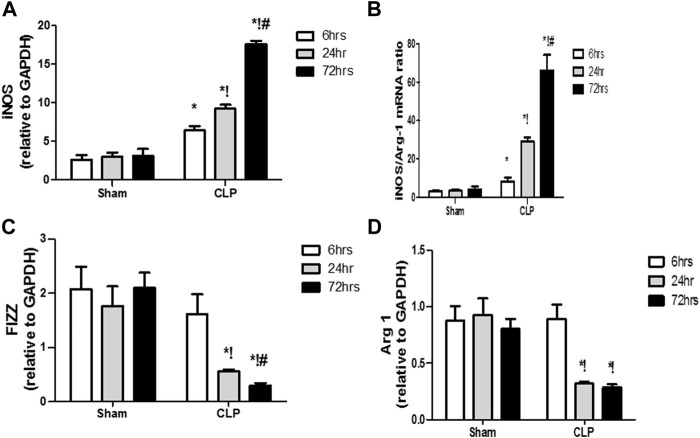
Changes in macrophage polarization over time in sepsis-induced encephalopathy. **(A–D)** Quantitative real-time reverse-transcription polymerase chain reaction analysis of macrophage polarization biomarkers: M1 pro-inflammatory macrophage iNOS **(A)** and M2 anti-inflammatory macrophage iNOS/Arg-1 ratio **(B)**, FIZZ1 **(C)**, and Arg-1 **(D)**. All expression levels were normalized to that of GAPDH. Data are the means ± SEM. *p < 0.05 vs. the corresponding sham group; !*p* < 0.05 vs. the 6 h CLP group; ^#^ *p* < 0.05 vs. the 24 h CLP group. *N* = 10 rats per group.

**FIGURE 7:**
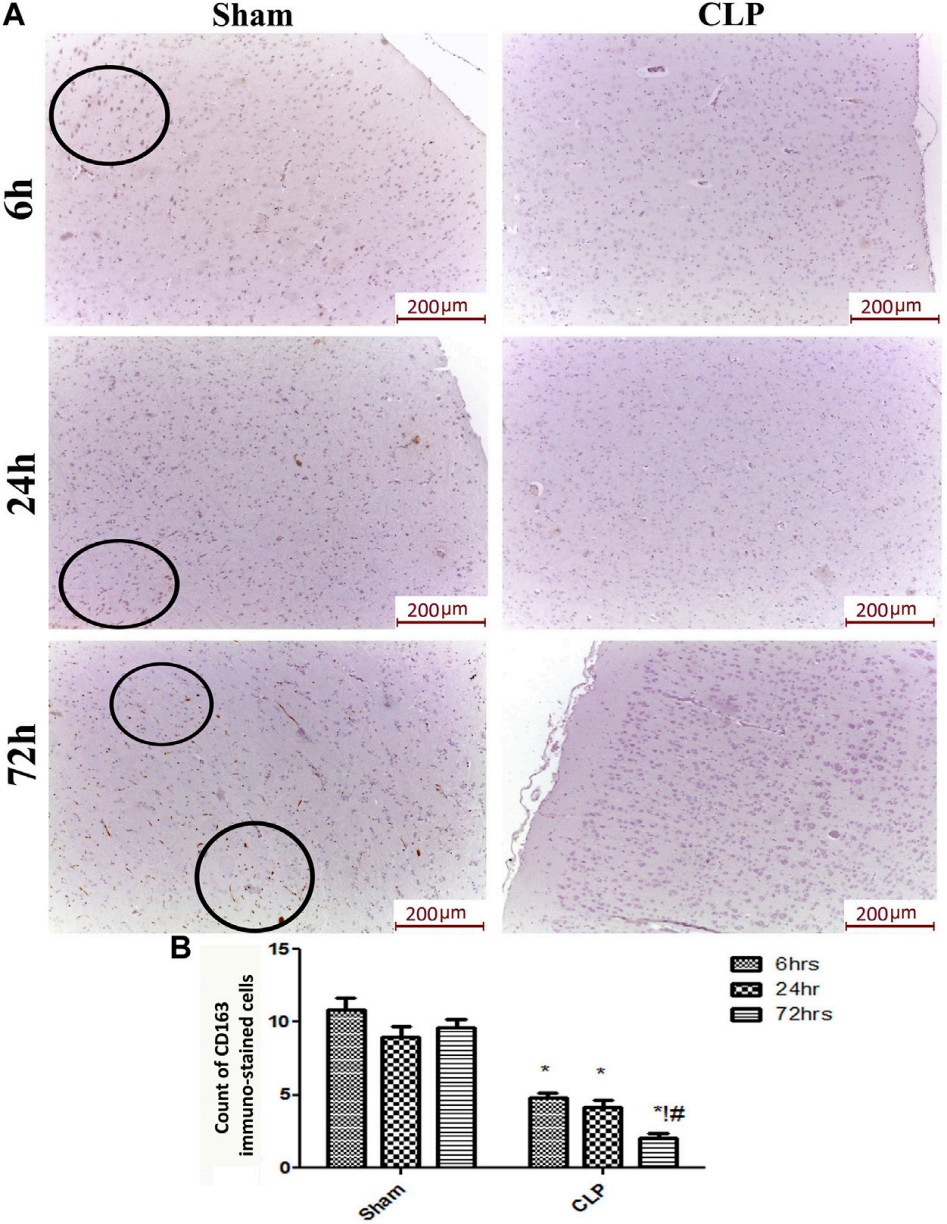
Sepsis mitigated the infiltration of CD 163 macrophages in brain tissues. **(A)** Representative images of immunohistochemical staining for CD163 in the sham and cecal puncture and ligation (CLP) groups at different time points. **(B)** Count of 163 immunohistochemically stained cells. Data are presented as the mean ± S.E.M. (*n* = 6). *: Statistically significant vs. the Sham; !:significant vs. 6 h CLP, #: significant vs. 24 h CLP, as assessed using ANOVA with the Bonferroni post-hoc test.

The microglia exist in two distinct phenotypes, namely the pro-inflammatory M1 phenotype and anti-inflammatory M2 phenotype, which acquire opposing functions. We assessed the immunohistochemical and gene expression analyses of the M1-pro-inflammatory markers (iNOS, CD68, and CD8) and M2-anti-inflammatory markers (Arg1, FIZZ1, and CD163) to distinguish the type of proliferating microglia in the histological sections (Figures 4–7). Significant increase in the count of CD 8 (Figures 4A,B), and CD 68 (Figures 5A,B) -positive cells along with upregulation of iNOS expression (Figure 6A) was observed at 6, 24 and 72 h after CLP. Moreover, the iNOS/arg-1 ratio increased significantly from 6 to 72 h post-CLP (Figure 6B). There were no significant differences in the expression of M2 phenotype biomarkers at 6 h post-CLP. At 24 h. We observed significant downregulation of FIZZ1 and Arg1 (Figures 6C,D), concomitant with a decrease in the count of CD 163-positive cells (Figures 7A,B), which was maintained for up to 3 days post-sepsis.

### Septic encephalopathy is significantly related to increased brain iron and not systemic iron *via* the reduction of iron-storage protein ferritin

Previous data clarifying microglial proliferation and the shift of microglia towards the pro-inflammatory phenotype essentially necessitate a parallel change in iron metabolism. This can be attributed to the role of microglia as cardinal cells in maintaining iron homeostasis in the brain. Under inflammatory conditions, microglia generally sequester iron from bacteria to limit their proliferation. Thus, we subsequently evaluated the brain level of iron and ferritin, the iron-storage protein, in addition to the serum iron-related markers [serum iron, Total Iron-Binding Capacity (TIBC), and transferrin saturation].

**FIGURE 8:**
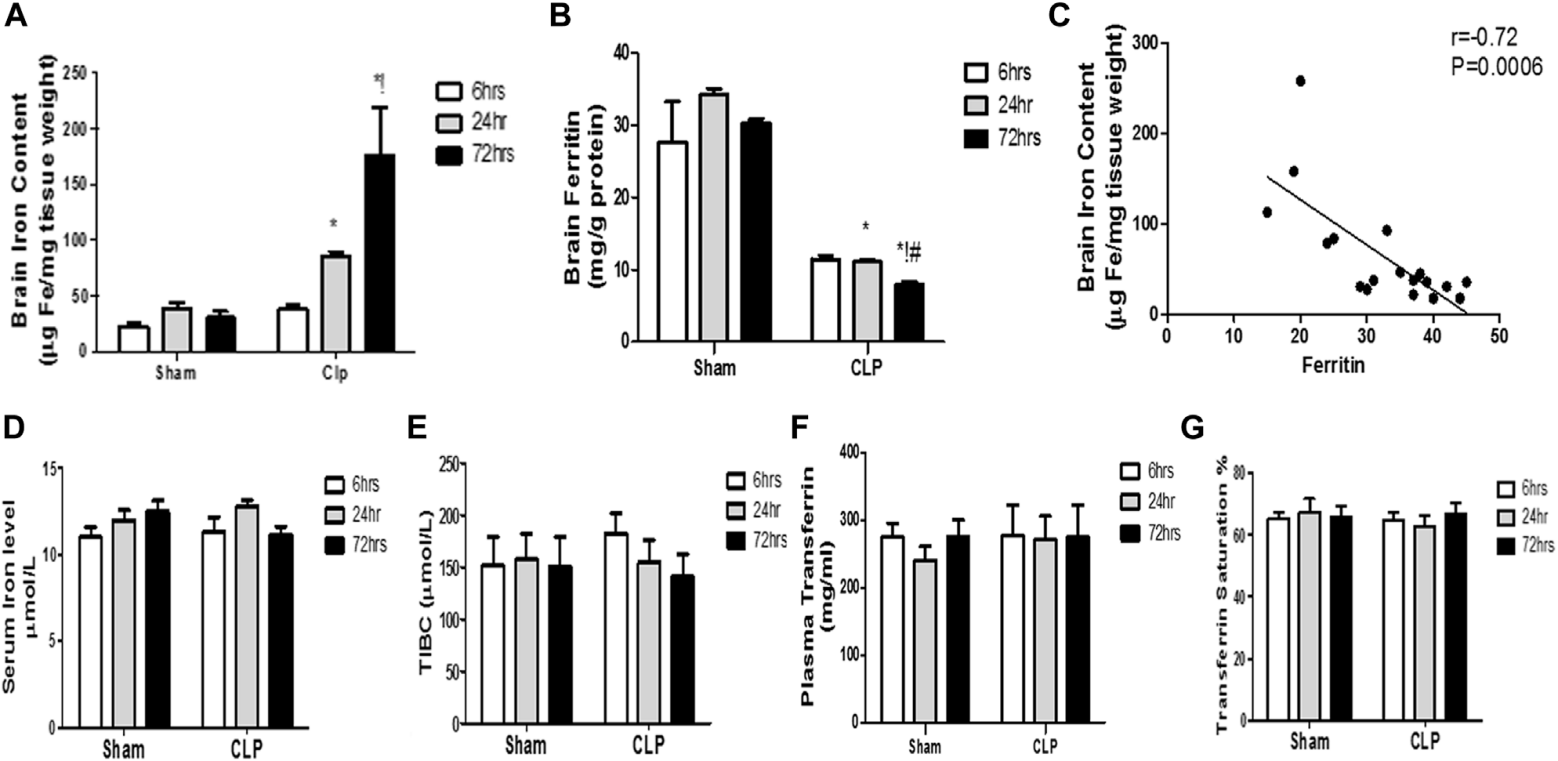
Changes in serum transferrin and brain ferritin over time during sepsis. The quantitative analysis of brain ferritin **(A)** and serum transferrin **(B)** showed a significant decrease in brain ferritin level, but not in serum transferrin, at 24 and 72 h after sepsis. **(C)** Pearson’s correlation analysis between iron level in the brain and ferritin level. The serum iron level **(D)**, the total iron binding capacity (TIBC) **(E)**, the plasma transferrin **(F)**, and the percentage of transferrin saturation **(G)** are not significantly different between the sham- and CLP-operated groups. Data are the means ± SEM. *p < 0.05 vs. the corresponding sham group; !*p* < 0.05 vs. the 6-h CLP group; ^#^ *p* < 0.05 vs. the 24-h CLP group. *N* = 10 rats per group.

Our findings revealed significant increase in the brain iron concentration beginning at 24 h post-CLP, which remained considerably higher than those detected in the sham-operated group till 72 h following sepsis induction (Figure 8A).

By contrast, the ferritin content of the brain began to significantly decrease 24 h post-CLP, with the most substantial reduction observed at 72 h post-CLP (Figure 8B).

Moreover, we observed a strong negative correlation between the iron content of the brain and ferritin levels (Figure 8C).

This decrease in brain ferritin levels can explain the excess free iron burden of the brain. This is because iron is intra-cellularly stored as ferritin. The impairment of iron storage *via* ferritin essentially contributes to free iron toxicity. By contrast, the serum iron, TIBC, plasma transferrin, and transferrin saturation were not significantly different between the sham- and CLP-operated groups, and remained constant during the 72 h of the experiment (Figures 8D–G).

### Changes in the time course in the two iron uptake systems in septic encephalopathy

The modulation of the transcription of iron transport and uptake genes is another crucial mechanism that defines the involvement of pro-inflammatory microglia in iron dyshomeostasis. Thus, we evaluated sepsis-induced changes of the two cardinal iron uptake systems, namely, the transferrin- bound iron system (TBI) (composed of the transferrin receptor protein 1 (TfR-1) and cubilin) and the non-transferrin-bound iron system (NTBI) (composed of the divalent metal transporter 1 (DMT-1) and zinc transporters (ZIPs)).

**FIGURE 9:**
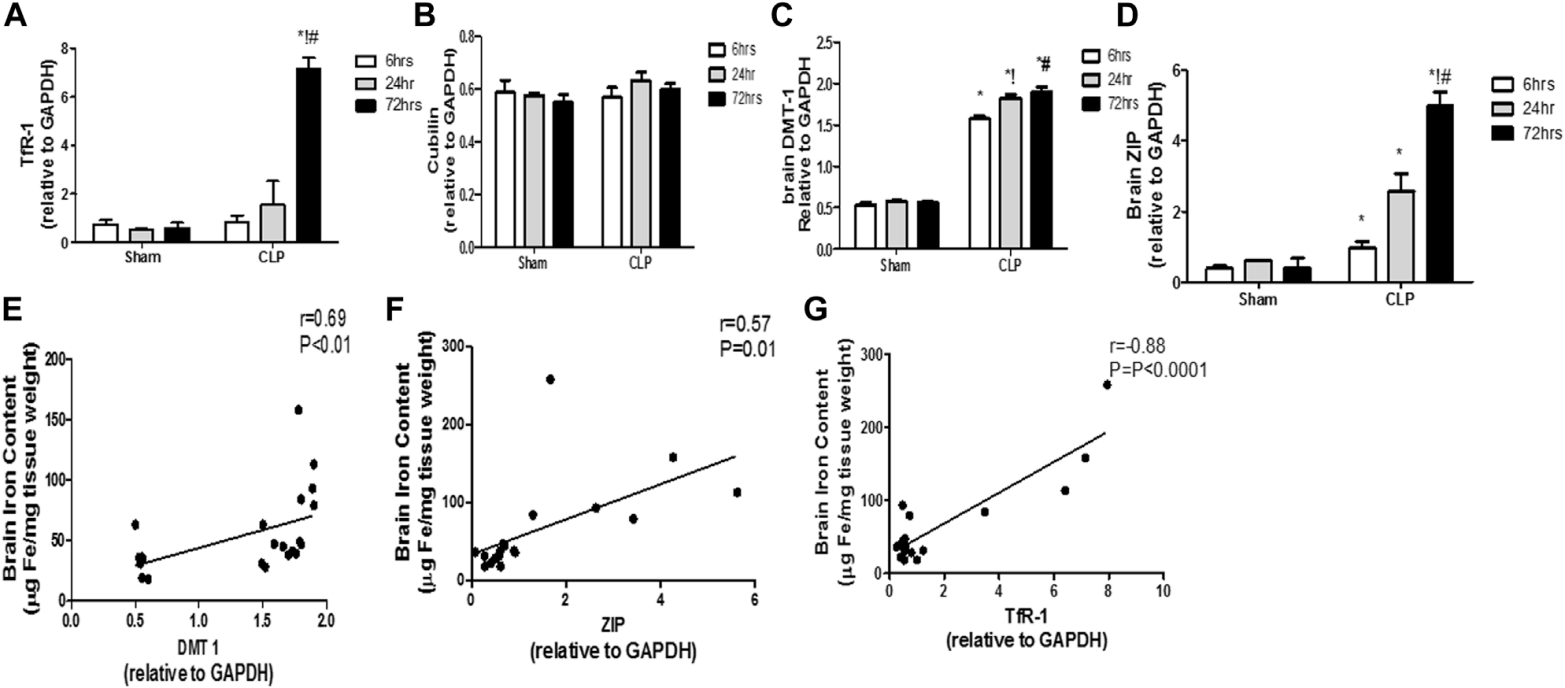
Transferrin- and non-transferrin-bound iron-uptake gene expression in the brain during sepsis. **(A–D)** Quantitative real-time reverse-transcription polymerase chain reaction analysis of the transferrin receptor protein 1 (TfR-1) **(A)**, cubilin **(B)**, the divalent metal transporter 1 (DMT-1) **(C)**, and the brain zinc transporters (ZIP) **(D)**. The expression levels of transferrin-bound iron transporters (TBI) (TfR-1**(A)** and cubilin **(B)**) in the brain during sepsis were not altered, whereas sepsis increased the expression of NTBI transporters (DMT-1**(C)** and ZIP **(D)**). **(E–G)** Pearson’s correlation analysis between the iron level in the brain and the DMT-1 **(E)** the ZIP **(F)** and the TfR-1 **(G)**. Data are the means ± SEM. *p < 0.05 vs. the corresponding sham group; !*p* < 0.05 vs. the 6 h CLP group; ^#^ *p* < 0.05 vs. the 24 h CLP group. *N* = 10 rats per group.

The TBI transporters revealed differential response to sepsis. Moreover, the brain TfR-1 expression was significantly increased at 72 h post-CLP, whereas cubilin expression was not altered, compared with the corresponding sham groups (Figures 9A,B). Contrarily, the NTB expression was significantly increased 6 h post-CLP, and remained significantly higher than those of the sham-operated animals at 24 and 72 h post-CLP (Figures 9C,D).

These data highlight the special role of an upregulated NTBI system with septic encephalopathy in the promotion of free iron toxicity, unlike the almost stable expression of TBI.

We observed a strong linear relationship between the changes in TfR-1 and brain iron content. Furthermore, we observed a positive correlation between NTBI expression and brain iron levels (DMT-1: *r* = 0.69; *p* < 0.0001; ZIP: *r* = 0.57; *p* < 0.01), thereby providing further evidence for their simultaneous roles in brain iron dyshomeostasis (Figures 9E–G).

### High iron levels in the brain tissue are associated with M1 polarization

**FIGURE 10:**
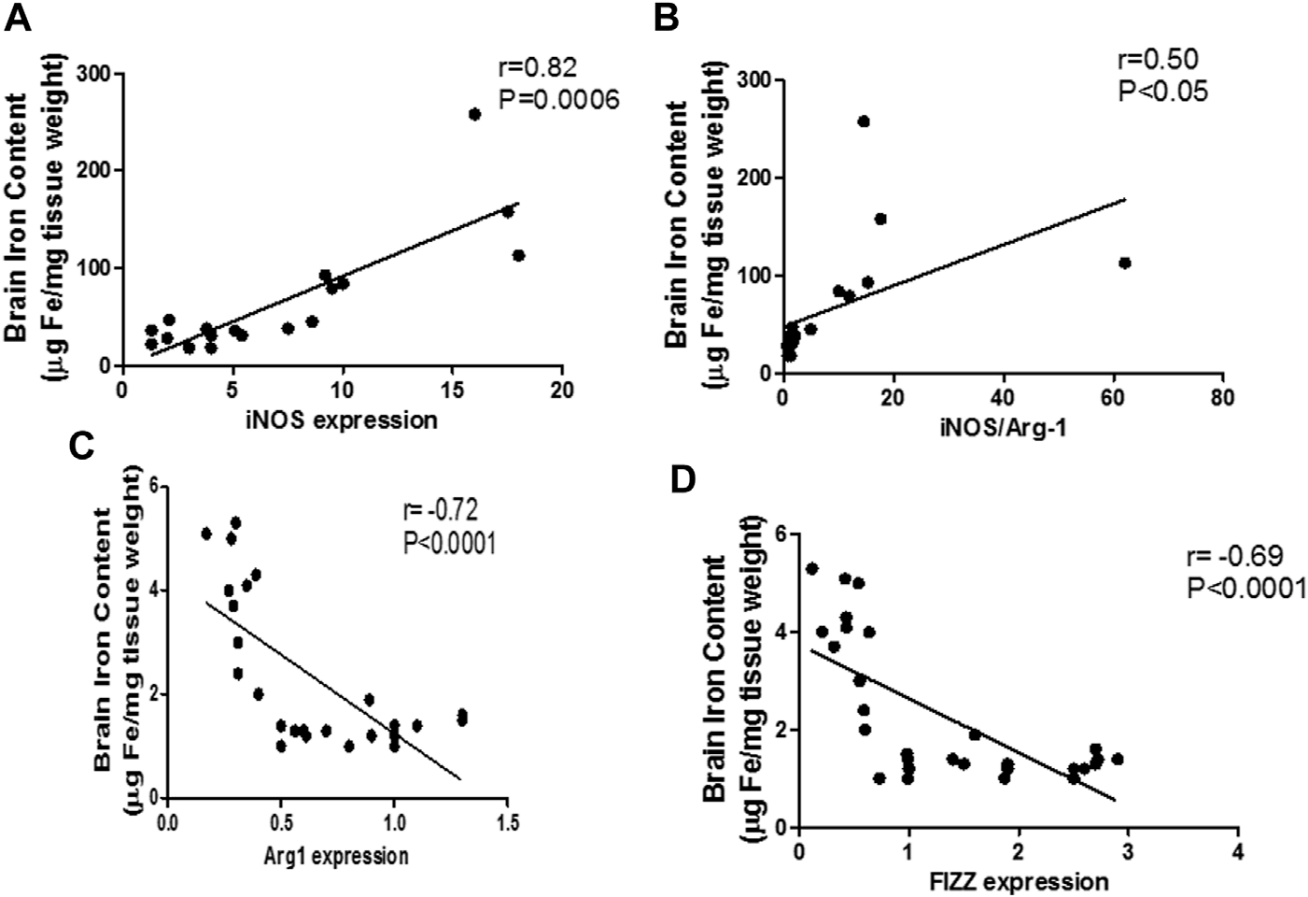
Linear regression analysis between brain iron levels and pro-inflammatory macrophage (M1) polarization markers. Significant positive correlation is noticed between the brain iron levels and the pro-inflammatory markers: iNOS **(A)** and iNOS/Arg-1 ratio **(B)**. Significant negative correlation is noted between brain iron level and the anti-inflammatory markers Arg-1 **(C)** and FIZZ **(D)**.

The mentioned interplay between pro-inflammatory microglia and iron dyshomeostasis was further confirmed through linear regression analysis curves, which revealed a significant linear relationship between the M1 markers and iron levels during sepsis in the brain (iNOS: *r* = 0.82; *p* < 0.0001; iNOS/arg-1: *r* = 0.84; *p* < 0.0001) (Figures 10A,B). Furthermore, we identified a moderate negative relationship between M2 markers and the brain iron content (arg-1: *r* = −0.72; *p* < 0.0001, FIZZ: *r* = −0.69; *p* < 0.0001) (Figures 10C,D).

## Discussion

Our data from CLP-induced encephalopathy established an important role for NTBI upregulation in parallel with ferritin reduction in iron dyshomeostasis, which occurred after microglial activation and polarization toward pro-inflammatory (M1)-type activity, which was not adequately balanced by the anti-inflammatory (M2) phenotype and coincided with significant neurological function impairment and neuronal loss.

Encephalopathy is a frequent and reversible complication of sepsis, which is characterized by persistent sickness behavior and cognitive impairment. Consistent with previous findings, the present study revealed the presence of cognitive and sensorimotor dysfunctions, which are believed to be caused by neuroinflammation within the frontal cortex and hippocampus (Vilar-Pereira et al., 2015; Mohamed et al., 2019).

The iron level in the brain increased concomitantly with the increase in neurodegeneration and impaired neurological function. Iron overload within the brain, rather than systemically, seems to partially contribute to the progression of CLP-induced encephalopathy. A growing body of evidence suggests that free-iron toxicity contributes to the pathogenesis of neurodegenerative diseases (Carocci et al., 2018; Zhang et al., 2015). Excessive labile iron promotes the formation of free radicals, lipid peroxidation, and eventually neuronal death (Daglas and Adlard, 2018). However, the mechanism underlying iron dyshomeostasis has not been established.

Among brain cells, microglia are believed to be responsible for maintaining iron homeostasis in the brain (Gong et al., 2019). The iron released by heme catabolism and transported to the brain is sequestered in microglia (McCarthy et al., 2018a; Nnah and Wessling-Resnick, 2018). Consistent with the results of Michels et al. (2020), we found that early activation of M1 microglia was followed by the predominance of the M1 over the M2 phenotype. Distinct phenotypes acquire opposing functions under the influence of different micro-environmental cues. The predominance of M1-phenotype cells worsens the neuronal injury after sepsis, because the attenuation of the M2 anti- inflammatory activation causes impaired neuronal repair (Michels et al., 2020). Early M1 phenotype activation is important for brain protection, whereas long-term activation of the M1 phenotype without counterbalancing of the M2 phenotype may lead to the aggravation of CLP-associated encephalopathy. This was further reinforced by the fact that neuronal degeneration occurs concomitantly with the persistent elevation of the M1 cytokines (IL1 and TNF), coinciding with the reduction of the M2 pro-resolution cytokines (IL-10 and IL-4) (Michels et al., 2020; D’Avila et al., 2018; Michels et al., 2019). Microglia were recently recognized as important regulators of iron homeostasis. However, a systems-level understanding of how different polarizations reshape the iron storage and transport system warrants further investigation.

We suggest that the microglia polarized to the pro-inflammatory activation states contribute to changes in the transcription of iron transport and storage genes. The causative role of microglia polarization in sepsis-associated iron dyshomeostasis is supported by evidence that microglial cells can acquire iron *via* the lipopolysaccharide (LPS)-induced upregulation of iron transport genes, and that microglial polarization and iron uptake are strongly correlated (McCarthy et al., 2018a).

We found an interplay between NIBT systems and ferritin levels, with consequent alteration in the iron levels. In contrast with the almost stable expression of TBI, NTBI expression was significantly increased in parallel with the sepsis-associated iron overload. Accordingly, the NTBI uptake system participated significantly in the labile iron overload in immortalized microglial (IMG) cells treated with lipopolysaccharide, whereas IMG cells acquired iron *via* TBI when cultured with the anti-inflammatory interleukin 4 (McCarthy et al., 2018a; Nnah and Wessling-Resnick, 2018). Furthermore, the sex differences in immune responses depend on sex-biased gene expression, mainly of genes associated with iron-uptake systems. Immune cells sequester iron to deprive the pathogens from iron, thus limiting their proliferation (Xu et al., 2020; Kezele and Ćurko-Cofek, 2020; Sharawy et al., 2011).

The differences in iron homeostasis observed for the various microglia phenotypes can be relevant for microglial metabolic changes, eventually favoring the generation of an antimicrobial environment (Recalcati and Cairo, 2021); (Recalcati et al., 2019). Microglia reprogram their metabolism to shift toward glycolysis, rather than oxidative phosphorylation, in response to Toll-like receptor activation. Scanty *in vitro* evidence suggested that invading pathogens may alter the brain microenvironment, thus inducing changes in iron uptake by microglial cells. The M1 phenotype may promote the internalization of iron, whereas the M2 phenotype is responsible for iron release (Di Paola et al., 2021). Moreover, infection may alter the microglial metabolism and the requirements for iron. Microglia sequester iron in an effort to deprive the invading bacteria of this essential element, thus limiting infection (McCarthy et al., 2018a). Under pro-inflammatory conditions, increased glycolysis and acidification seem to affect the iron uptake by microglia through the activation of the iron regulatory protein 1 (IRP1) (which is an RNA-binding protein that regulates the synthesis of iron storage and -transport proteins) (Xu et al., 2018). Moreover, polarization to an anti-inflammatory state was associated with arginase 1 upregulation and increased nitric oxide production, which could support mitochondrial respiration and limit the glycolytic response and acidosis, subsequently resulting in a decrease in iron uptake and increase in iron efflux (Carr et al., 2020; McCarthy et al., 2018b).

Iron status is also critically determinant of IL-1β and TNF-α release from microglia through the modulation of NF-κB and mitochondrial OXPHOS (Xia et al., 2021; Daglas and Adlard, 2018), thus creating a positive feedback loop that leads to chronic neuroinflammation (Di Paola et al., 2021; Zhu et al., 2015; Mertens et al., 2021). *In vitro*, the transformation of macrophages into the M1 phenotype can be accelerated by the presence of iron. In a murine model of chronic venous leg ulcers, an abundant deposition of iron in macrophages was found to induce macrophages with a pro-inflammatory phenotype, which impaired wound healing (Sindrilaru et al., 2011). In atherosclerotic plaque, iron overload was found to polarize macrophages toward the M1 phenotype, thus leading to progression of the disease (Sindrilaru et al., 2011; Kamanna et al., 2012). Furthermore, sequestration of iron *via* ferritin overexpression in macrophage suppressed LPS-induced cytokines (Fan et al., 2014). Thus, targeting free iron may present a novel approach for shifting macrophages toward the anti-inflammatory phenotype.

As a major iron storage, ferritin adversely impacts the progression of several neurological disorder, such as Alzheimer’s disease (Filosto et al., 2018) and cerebral small vessel disease (Gong et al., 2019). In our study, ferritin levels were also significantly reduced, an observation that potentially explains the free iron overload. In the iron-overload status, loss of iron-buffering capacity seems to decrease the tolerance of the brain to sepsis. At a high level of iron, ferritin was found to be decreased because of its breakdown as a consequence of iron-dependent oxidative stress. Oxidative stress enhances ferritin degradation *via* the lysosomal and proteasome pathways, mediated by nuclear receptor coactivator 4 (NCOA4) activation (Gong et al., 2019; Arosio et al., 2017). Furthermore, treatment of human macrophages with cytokines decreased the total IRP activity, thus decreasing the ferritin content (Recalcati et al., 2010).

Septic-associated encephalopathy is characterized by crucial neurological changes with potential long-term psychocognitive disorders (Semmler et al., 2013; Mazeraud et al., 2020) where attention, verbal fluency, executive function, verbal memory, and quick mental processing are the main cognitive functions impaired, whereas visual memory and visuoconstructive ability are usually spared (Pandharipande et al., 2013).

The psychological disorders implicated in sepsis-induced encephalopathy also include anxiety, depression and post-traumatic stress disorder (Lund-Sørensen et al., 2016; Righy et al., 2019). These psychological and cognitive disorders dramatically impact quality of life and functional status (Herridge et al., 2003).

Together with previous studies (Recalcati et al., 2010), our data endorse that iron accumulation and microglia polarization are intimately linked. Therefore, to mitigate neuroinflammation, it would be crucial to develop a combination therapeutic approach using both anti-inflammatory and iron-chelator drugs (Lehmann et al., 2015). In recent years, there has been a growing interest in the development of multi-target drugs that combine iron-chelation and anti-inflammatory properties. Recent studies of patients with Parkinson’s disease treated with deferiprone showed decreases in substantia nigra iron content and improved scores on the Parkinson’s Disease Rating Scale (Ward et al., 2021). Using a septic animal model, Fokam et al. (2020) reported that the iron-chelator DIBI could be a promising anti-inflammatory treatment, either alone or combined with antibiotics.

We believe that our current study could pave the way to implement the iron-chelating agents as an adjunctive line of treatment for improvement of the outcome of sepsis-associated encephalopathy and alleviation of the long lasting post-sepsis symptoms.

## Conclusion

Taken together, our results suggest that microglia regulate the iron-uptake pathway to adapt to the changes in energy metabolism elicited by M1/M2 polarization. Microphage polarization differentially affected the utilization of the NTBI or TBI pathways, with predominance of NTBI under pro-inflammatory conditions. The shift in iron transport and the downregulation of ferritin are strongly attributed to the persistence of neuroinflammation. Targeting free iron may provide a novel approach for skewing microglia toward the anti-inflammatory phenotype.

## Data Availability

The original contributions presented in the study are included in the article/Supplementary Material, further inquiries can be directed to the corresponding author.
