# Supplementary material for: Iron dyshomeostasis and time-course changes in iron-uptake systems and ferritin level in relation to pro-inflammatory microglia polarization in sepsis-induced encephalopathy
Source: Front Physiol. 2022 Aug 12;13:953206. doi: 10.3389/fphys.2022.953206 (PMC9413069; doi:10.3389/fphys.2022.953206)
Supplement: Supplementary file 1 [file DataSheet1.docx]

**Supplement file 1**

**Table 1. Criteria for severity grading of sepsis using Murine Sepsis Score (MSS) in an experimental model of cecal ligation and puncture**

| **Variable** | **Score and description** |
| --- | --- |
| **Appearance** | 0- Coat is smooth  1- Patches of hair piloerected  2- Majority of back is piloerected  3- Piloerection may or may not be present, mouse appears “puffy”  4- Piloerection may or may not be present, mouse appears emaciated |
| **Level of consciousness** | 0- Mouse is active  1- Mouse is active but avoids standing upright  2- Mouse activity is noticeably slowed. The mouse is still ambulant.  3- Activity is impaired. Mouse only moves when provoked, movements have a tremor  4- Activity severely impaired. Mouse remains stationary when provoked, with possible tremor |
| **Activity** | 0- Normal amount of activity. Mouse is any of: eating, drinking, climbing, running, fighting  1- Slightly suppressed activity. Mouse is moving around bottom of cage  2- Suppressed activity. Mouse is stationary with occasional investigative movements  3- No activity. Mouse is stationary  4- No activity. Mouse experiencing tremors, particularly in the hind legs |
| **Response to stimulus** | 0- Mouse responds immediately to auditory stimulus or touch  1- Slow or no response to auditory stimulus; strong response to touch (moves to escape)  2- No response to auditory stimulus; moderate response to touch (moves a few steps)  3- No response to auditory stimulus; mild response to touch (no locomotion)  4- No response to auditory stimulus. Little or no response to touch. Cannot right itself if pushed over |

**Table 1. Criteria for severity grading of sepsis using Murine Sepsis Score (MSS) in an experimental model of cecal ligation and puncture (cont)**

| **Variable** | **Score and description** |
| --- | --- |
| Eyes | 0- Open  1- Eyes not fully open, possibly with secretions  2- Eyes at least half closed, possibly with secretions  3- Eyes half closed or more, possibly with secretions  4- Eyes closed or milky |
| Respiration rate | 0- Normal, rapid mouse respiration  1- Slightly decreased respiration (rate not quantifiable by eye)  2- Moderately reduced respiration (rate at the upper range of quantifying by eye)  3- Severely reduced respiration (rate easily countable by eye, 0.5 s between breaths)  4- Extremely reduced respiration (>1 s between breaths) |
| Respiration quality | 0- Normal  1- Brief periods of laboured breathing  2- Laboured, no gasping  3- Laboured with intermittent gasps  4- Gasping |
